# Biowaxes from Palm Oil as Promising Candidates for Cosmetic Matrices and Pharmaceuticals for Human Use

**DOI:** 10.3390/ma16124402

**Published:** 2023-06-15

**Authors:** Laura María Chaparro, Laura Fernanda Neira, Daniel Molina, Diego Rivera-Barrera, Maribel Castañeda, Luis Javier López-Giraldo, Patricia Escobar

**Affiliations:** 1 Centro de Investigación de Enfermedades Tropicales (CINTROP-UIS), Departamento de Ciencias Básicas, Escuela de Medicina, Universidad Industrial de Santander, Bucaramanga 680002, Colombia; 2 Laboratorio de Resonancia Magnética Nuclear, Escuela de Química, Universidad Industrial de Santander, Bucaramanga 680002, Colombia; 3 Centro de Innovación y Tecnología—ICP-ECOPETROL S.A, Bogotá 110911, Colombia; 4 Grupo de Investigación en Ciencia y Tecnología de Alimentos—CICTA, Escuela de Ingeniería Química, Universidad Industrial de Santander, Bucaramanga 680002, Colombia

**Keywords:** palm oils, biowaxes, safety, cytotoxicity, SEM, FTIR, UV–Vis, ^1^H NMR

## Abstract

The production of waxes from vegetable oils, such as palm oil, for use as a base material in products for human applications is an alternative to those derived from petroleum and animals. Seven palm oil–derived waxes, called biowaxes (BW1–BW7) in this work, were obtained by catalytic hydrotreating of refined and bleached African palm oil and refined palm kernel oil. They were characterized by three properties: compositional, physicochemical (melting point, penetration value, and pH), and biological (sterility, cytotoxicity, phototoxicity, antioxidant, and irritant). Their morphologies and chemical structures were studied by SEM, FTIR, UV–Vis, and ^1^H NMR. The BWs presented structures and compositions similar to natural biowaxes (beeswax and carnauba). They had a high concentration of waxy esters (17%–36%) with long alkyl chains (C, 19–26) per carbonyl group, which are related to high melting points (<20–47.9 °C) and low penetration values (2.1–3.8 mm). They also proved to be sterile materials with no cytotoxic, phototoxic, antioxidant, or irritant activity. The biowaxes studied could be used in cosmetic and pharmacological products for human use.

## 1. Introduction

The term wax is used to describe certain solid organic materials with melting points between 45 and 100 °C, which melt without decomposing and are hydrophobic but soluble in organic solvents [1]. They are obtained semi or synthetically from plants, animals, and minerals. Approximately half of the world’s consumption of waxes is for the manufacture of candles, followed by packaging materials, corrugated cardboard, coatings, food, cosmetics, and pharmaceuticals, among others [1]. World production of industrial waxes amounts to 4.8 million tons ($6.7 billion), with petroleum waxes accounting for 85–90% of global consumption [2]. Natural waxes derived from plants or animals such as beeswax, carnauba, and candelilla are renewable, biodegradable, and non-toxic, and have excellent physicochemical properties. However, their cost is increasing due to supply shortages and the collapse of bees due to infection or exposure to agrochemicals [3,4].

Methods have been implemented to obtain biowaxes (BW) by structurally modifying the triglycerides (TG) of vegetable oils [5,6,7]. Vegetable oils from oleaginous crops such as sunflower, soybean, and African palm are mainly composed of TG, e.g., palmitic acid (16:0), stearic acid (18:0), oleic acid (18:1), linoleic acid (18:2), and α-linolenic acid (18:3), as well as a small amount of di- and mono-glycerides [8]. Through hydrogenation, esterification, interesterification, and transesterification reactions, as well as ester–alcohol reactions in the presence of chemical or enzymatic catalysts, materials with a wide range of physical properties useful for the production of fuels (biodiesel) and oleochemicals are generated [5,9,10,11].

The oil obtained from the *Elaeis guineensis* palm has served as a substrate for obtaining waxy compounds (biowaxes) similar in composition to beeswax, carnauba, and spermaceti, and it possesses beneficial properties (i.e., emollients, emulsifiers, and biological) for use in the cosmetic, pharmaceutical, and food industries [5,6]. By catalytic hydrotreating refined palm oil, it has been possible to obtain biowaxes composed of waxy esters such as hexadecanoyl hexadecanoate (cetyl palmitate, C_32_H_64_O_2_) and octadecyl octadecanoate (stearyl stearate, C_36_H_72_O_2_), in addition to hydrocarbons, fatty alcohols (octadecanol, C_18_H_38_O, and hexadecanol, C1_6_H_34_O), fatty acids, and smaller amounts of tri-, di-, and mono-glycerides [12].

Some natural waxes—such as carnauba, beeswax, and candelilla— are biocompatible (inert), non-toxic materials that the Food and Drug Administration (FDA) has generally recognized as safe (GRAS) for use as food additives and as components in cosmetics and pharmaceutical products [13,14,15]. They are promising candidates for developing particles as drug delivery systems, for substituting solid fats with oleogels in certain food products (chocolate cream and types of butter), and as a component of lipid-based nanocarriers in cosmetics [14,15,16,17,18]. Agencies exist that periodically review and evaluate the safety of waxes (or their main components, i.e., waxy esters) for human use, and some tests result in warnings about the (eco)toxicological risks that they pose. This must be considered before manufacturing products [18,19,20,21]. It is known that fatty acids derived from palm oils are safe, as are certain fatty acid esters produced with palmitic or stearic acid and alcohol, and expert Cosmetic Ingredient Review (CIR) panels have considered them to be suitable for use in cosmetics [22,23]. However, to determine that other biowaxes are safe components in cosmetics or drugs, it is important to ensure that they do not produce adverse reactions such as irritation, eczema, or allergic contact dermatitis [24,25].

In this work, seven biowaxes (BW1–BW7) were obtained from refined and bleached palm and palm kernel oils by means of different hydrotreating conditions. Their potential use in the manufacture of products for human consumption was evaluated based on the characterization of three properties: physical, chemical, and biological.

## 2. Materials and Methods

### 2.1. Biowaxes (BWs)

Biowaxes were obtained following the methodology described in Guzman et al., 2010 and 2013 [9,12], modifying the reaction conditions and catalysts used according to Olarte et al., 2023 [26], as follows: Biowaxes BW1–BW7 were obtained in a pilot-scale fixed-bed reactor using a nickel molybdenum catalyst (NiMo/Al_2_O_3_), with temperatures ranging from 240 to 260 °C, pressures between 800 and 1300 psig, and Liquid Hourly Space velocity (LHSV) of 1 to 2 h^−^^1^. BW1–BW5 were obtained by catalytic hydrotreating refined palm oil (RPO), BW6 was obtained by catalytic hydrotreating refined palm kernel oil (RPKO), and finally, BW7 was obtained from bleached palm oil (BPO). Biowaxes BW1–BW7 were selected from a set of 45 previously obtained biowaxes based on descriptive and physicochemical analyses [27], contamination by microorganisms, and comparison of catalytic systems. Some experiments used natural commercial beeswax and carnauba wax as controls.

### 2.2. Organoleptic and Physicochemical Characteristics

The color, odor, and consistency of the seven biowaxes (BW1–BW7) were determined. To determine the pH, 100 mg of BW was incubated in 1 milliliter of sterile distilled water at room temperature for 20 days. A digital pH meter was used to measure the pH of the supernatants after 5, 10, and 20 days of incubation. Measurements were performed in duplicate. The melting point (MP) was determined according to NTC 213:2013. The melted BWs were placed in capillary tubes that were sealed and left at room temperature for 24 h until solidification. They were then placed in a melting point apparatus (Hanon MP-420, Hanon Group, Jinan, China) to determine the temperature (°C) at which the BWs slid down the capillary. The penetration value (PV) or consistency was determined according to ASTM D1321. The melted BWs were placed in the equipment’s container, and after cooling, they were put in a penetrometer (penetrometer PNR 12, Anton Paar, Ashland, VA, USA) composed of a 150-g cone with a needle that was allowed to sink into the sample for 5 s, at a temperature of 25 °C. Penetration was recorded as the depth, in tenths of a millimeter (penetration unit, PU), to which the cone sank into the biowaxes. The greater the depth, the softer the material.

### 2.3. Chemical Composition of the Palm Oil Biowaxes

Two milligrams of each of the biowax samples that were obtained by hydrotreating the palm or palm kernel oil were diluted in carbon disulfide (2:98), after which 0.1 microliters of the solution was injected into a gas chromatography-mass spectrometry equipped with a flame ionization detector (GC-MS-FID) through a high-temperature programmable injection port (Agilent, Santa Clara, CA, USA). The components were separated with an Agilent DB-HT column operating in the range of 40 to 400 °C. MSD Chemstation E.02.01 software and SIMDIS EXPERT MS v10 were used to determine and quantify the main families, i.e., kerosenes, fatty acids, fatty alcohols, waxy esters, and acylglycerides. The results were expressed as the average of three measurements of the g/100 g sample.

### 2.4. Scanning Electron Microscopy (SEM)

An FEI Quanta FEG (Field Emission Gun 650, FEI Company, Hillsboro, OR, USA) scanning electron microscope equipped with energy dispersive x-ray spectroscopy (EDS) was used to observe the morphology and surface elemental composition of the different biowaxes. High vacuum images were taken with an accelerating voltage of 15 kV.

### 2.5. Fourier Transform Infrared Spectroscopy (FTIR)–ATR

The infrared (IR) spectra of the BWs were obtained using an FTIR spectrometer (Nicolet iS50 Thermo Scientific, Waltham, MA, USA). The samples were placed on the crystal, and the spectra were obtained by measuring the absorbance in the range of 400 to 4000 cm^−^^1^, with a 4-cm^−^^1^ resolution in the attenuated total reflectance (ATR) mode.

### 2.6. Ultraviolet-Visible Spectroscopy (UV–Vis)

The biowaxes were diluted in spectroscopic-grade toluene at a concentration of 10 mg/mL. Absorption spectra were recorded in the UV–Vis range (200 to 800 nm) at room temperature using a MultiSkan Go spectrophotometer (Thermo Fisher Scientific, Waltham, MA, USA).

### 2.7. Nuclear Magnetic Resonance (NMR) Spectroscopy

The biowaxes (30–50 mg) dissolved in 630 µL of deuterated chloroform (CDCl_3_) with 0.03% (*v*/*v*) tetramethylsilane (TMS) were transferred to 5-mm NMR tubes. Acquisition of the spectra was performed with a Bruker Avance III 400-MHz (9.4-T) spectrometer using a Zg30-pulse sequence with 32 scans, relaxation time (D1) of 10 s, receiver gain (RG) of 101, and spectral width (SWH) of 6009.615 Hz for the ^1^H NMR spectra. The spectra were processed with Mestre Nova 12.0 software, and the TMS signal at 0 ppm was used for proton scale fitting. The ^1^H NMR spectra were divided into 31 regions, and the percentage area of each proton type (${\%A}_{i})$ was calculated based on the equation $\%A_{i}=(A_{i}/A_{T})*100$, where $A_{i}$ is the area of the analyzed signal and $A_{T}$ is the total area of protons in the range of 0 to 10 ppm per spectrum.

### 2.8. Microbiological Test

The biowaxes (150 mg/mL) were incubated with 2 mL of brain heart infusion broth (BHI) for five days at 37 °C. Then, 100 µL were cultured on nutrient agar for 24 to 48 h at 37 °C for bacterial detection and on potato dextrose agar (PDA) for 5 to 7 days at 25 °C for fungal and yeast detection. The morphologies of the colonies were described, and the results were expressed in colony-forming units (CFU)/mL. Gram staining was performed to describe the type of bacteria in the positive samples.

### 2.9. Cell Lines

All the lines that were used were purchased commercially from ATCC, Manassas, VA, USA. Vero cells (ATCC-CCL81), a monkey kidney epithelial cell line, and human osteosarcoma HOS cells (ATCC-CRL1543) were cultured in an RPMI 1640 medium plus 10% hiFBS at 37 °C with 5% CO_2_ and 95% humidity. Fibroblast NIH/3T3 cells (ATTC CRL-1658) were cultured in a DMEM medium plus 5% hiFBS at 37 °C.

### 2.10. Toxicity Test on Cell Lines

The biowaxes (300 mg) and controls (non-waxes) were incubated with 2 milliliters of phosphate buffer saline (PBS) at a pH of 7.2 and a temperature of 37 °C. After 20 days, the supernatants were removed and stored at 4 °C until use. Vero cells (1 *×* 10^5^ cells/mL) and HOS cells (2.5 *×* 10^4^ cells/mL) were treated in triplicate for 72 h with 1:2 serial dilutions of the biowax supernatants, PBS or fresh culture medium (RPMI-1640 supplemented with 10% fetal bovine serum), at 37 °C with 5% CO_2_ and 95% humidity. Toxicity was determined by the 3-(4,5-dimethylthiazol-2-yl)-2,5-diphenyltetrazolium bromide (MTT) colorimetric method. Twenty µL of MTT (2.5 mg/mL) was added for 4 h, followed by 100 µL of dimethyl sulfoxide (DMSO). The reading was performed in a spectrophotometer (BioTek Synergy H1, Winooski, VT, USA) at 580 nm. Toxicity percentages were calculated according to the equation % cytotoxicity = (AB − AA)/AB × 100, where AB and AA are the absorbance values of the negative control and the sample, respectively. A heat map was used to express the results. Differences were analyzed with the student’s t-test. Values of *p* ≤ 0.05 were considered statistically significant.

### 2.11. Antioxidant Activity

The antioxidant activity was determined with the 2,2-diphenyl-1-picrylhydrazyl radical (DPPH) inhibition method. Serial dilutions of the biowaxes (0.5 to 500 mg/mL), ascorbic acid (0.001 to 1 mg/mL) (positive control), and methanol (negative control) were mixed with the DPPH radical (0.1 mg/mL) in a 1:10 ratio. After 20 min, absorbances were determined spectrophotometrically at 520 nm. The percent inhibition of the DPPH radical was calculated with the equation % inhibition DPPH = (AB − AA)/AB × 100, where AB and AA are the absorbance values of the negative control and the sample, respectively. Sigmoidal regression was used to determine the effective concentration of 50% (EC_50_).

### 2.12. Irritation Test (for Pharmaceutical Purposes Only)

A biowax formulation was prepared by mixing the biowax with mineral oil in a 1:2 ratio by heating and mixing using mechanical agitation (Ultra Turrex, IKA T10, IKA, Campinas, Brazil). That formulation will be used as a component of topical formulations for skin diseases: cream and solid lipid nanoparticles (SLNs) containing antiparasitic or antifungal drugs. BALB/c mice were used, which were kept in the Experimental Unit of the UIS Guatiguará Headquarters at 20 ± 3 °C with 12-h light/dark photoperiods, and food and water ad libitum. All procedures were performed following a protocol that was approved by the Ethics Committee of the Industrial University of Santander, Bucaramanga, Colombia, act N.2 from 21 February 2020. The backs of the mice were depilated, and 24 h later, they were treated topically with each formulation for 21 days. The degree of irritation was classified as follows: 0, no erythema; 1, mild erythema; 2, well-defined erythema; 3, moderate or severe erythema; and 4, severe erythema. The mice were sacrificed 15 days after completion of treatment with a mixture of ketamine + xylazine anesthetics administered intraperitoneally. Skin biopsies were collected and fixed with 10% formalin, embedded in paraffin, cut at 5 µm, and stained with hematoxylin-eosin. The intensity of the variables was classified as negative = 0, mild = +, moderate = ++, and severe = +++. They were analyzed by two experts.

### 2.13. Statistical Analysis

The Shapiro–Wilk test was used to evaluate the normality of the results. Using the statistical program GraphPad Prism 8.1 (GraphPad Software Inc., San Diego, CA, USA), the results between groups were compared using a one-way ANOVA test, along with post hoc comparisons (Tukey Dunnett’s test). A *p* < 0.05 was considered statistically significant.

## 3. Results and Discussion

### 3.1. Characteristics of the Biowaxes

The BWs were yellowish white and solid, except for BW6, which was liquid. The pH of the supernatants ranged from 4.4 to 5.4, with no differences during the various incubation days that were evaluated (Table 1). This result was important because we want to propose this material for human use in dermatology (pharmaceutical and cosmetic). Since the pH of the natural skin surface is acidic, on average 4.7, the pH obtained in BWs supernatants is potentially related to no or low risk of toxicological effects. The MP and PV of the BWs studied were similar (except BW6), with an average MP of 43.2 °C (standard deviation (SD) = 3.0) and a PV of 2.9 mm (SD = 0.6). The MP values of beeswax and carnauba wax were 62.2 and 81.6 °C, respectively, which were higher than those of the BWs studied. The PVs of beeswax and carnauba were 2.2 and 0.3 mm, respectively. BW1 penetration values were similar to those of beeswax (2.1 mm), while none of the BWs had as high of an MP or as low of a PV as those of carnauba, which were 81.6 °C and 0.3 mm, respectively. The MP and PV for refined palm oil (RPA) were 24.8 °C and 4.0 mm, respectively. Generally, PVs are correlated with the hardness of a wax; i.e., a higher PV indicates a lower hardness of the materials, and low penetration values are desired to achieve high firmness and good molding properties. For example, the BW1 sample presented a lower PV (2.1 mm) than the natural beeswax (2.2 mm), which is widely employed in the medicine, pharmaceutical, cosmetics, and food industries. Since the MP values of the BWs studied were lower than those of the natural waxes, crude biowaxes would have to be chemically modified or refined to achieve this property, when necessary.

### 3.2. Chemical Composition of the Biowaxes

Catalytic hydrotreating of palm oil produced BWs with paraffin percentages ranging from 13% to 40%, waxy esters (WE) from 16.7% to 35.6%, fatty alcohols (FA) from 2.1% to 13.5%, free fatty acids (FFA) from 8.8% to 27.4%, triglycerides (TG) from 11.8% to 28.6%, and mono- (MG) and di-glycerides (DG) under 1% (Table 2). BW1–BW5 were produced by hydrotreating refined palm oil (RPO) composed of free TG and FFA, with an MP of 24.8 °C and a PV of 4.0 mm, which presented WE percentages ranging from 23.7% and 35.6%, MP from 40 to 48 °C, and PV from 2.1 to 3.8. Using a catalytic process with refined palm kernel oil (RPKO) resulted in a liquid biowax (BW6) that presented the lowest WE value (16.70%) (Table 2). This study did not evaluate the initial composition of the RPKO oil. The compounds’ increased alkyl chain length that was generated (mainly WE) improved the physical properties of the material, such as cohesion, MP, and hardness [28].

### 3.3. Observations with Scanning Electron Microscopy (SEM)

**Figure 1:**
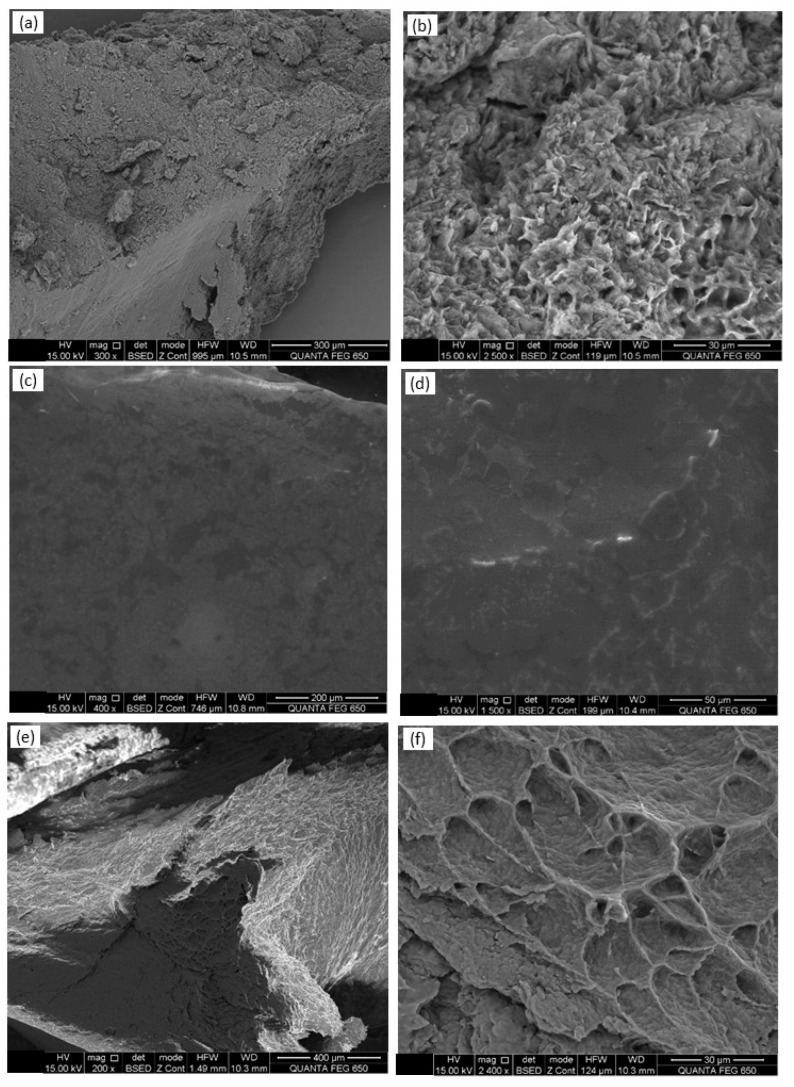
Scanning electron microscopy (SEM) images of biowaxes BW2 (**a**,**b**), BW6 (**c**,**d**), and beeswax (**e**,**f**), with two magnification values: 100 to 400× (right) and 1500 to 3000× (left). Figure S1 in the Supplementary Material presents the images of all the biowaxes.

At lower magnification, the SEM images of BW1–BW5 and BW7 showed irregular surfaces (smooth to rough), some of which were fractionated, with some differences between them (Figure S1a–p). The BW6 liquid presented a soft and smooth surface (Figure 1c,d). At higher magnifications, some of the biowaxes (i.e., BW2, Figure 1a,b) had a compact structure with sharp and well-defined angles and a uniform color, similar to what Trisnadewi et al. (2021) found for palm wax [30]. The EDS analysis (surface composition of the sample) could only be performed with BW2 since the others melted when coming into contact with the electron beam. This analysis resulted in carbon and hydrogen percentages of 87.59% and 12.41%, respectively, which are similar to those reported by other authors (82.33% and 17.67%, respectively) [30]. At 200×, the surface of the beeswax was composed of overlapping plates and white fibers that were distributed in the form of networks. At 2400×, a reticulated matrix of solid beeswax could be seen, with protruding ridges and valleys that formed asymmetrical pores. These structural characteristics are likely due to the amorphous and heterogeneous nature of the constituents (Figure 1e,f). At 100×, the carnauba wax presented a compact and non-porous structure, and at 3000×, there were randomly distributed lamellae with different sizes and shapes (Figure S1q,r).

### 3.4. FTIR–ATR Analysis

**Figure 2:**
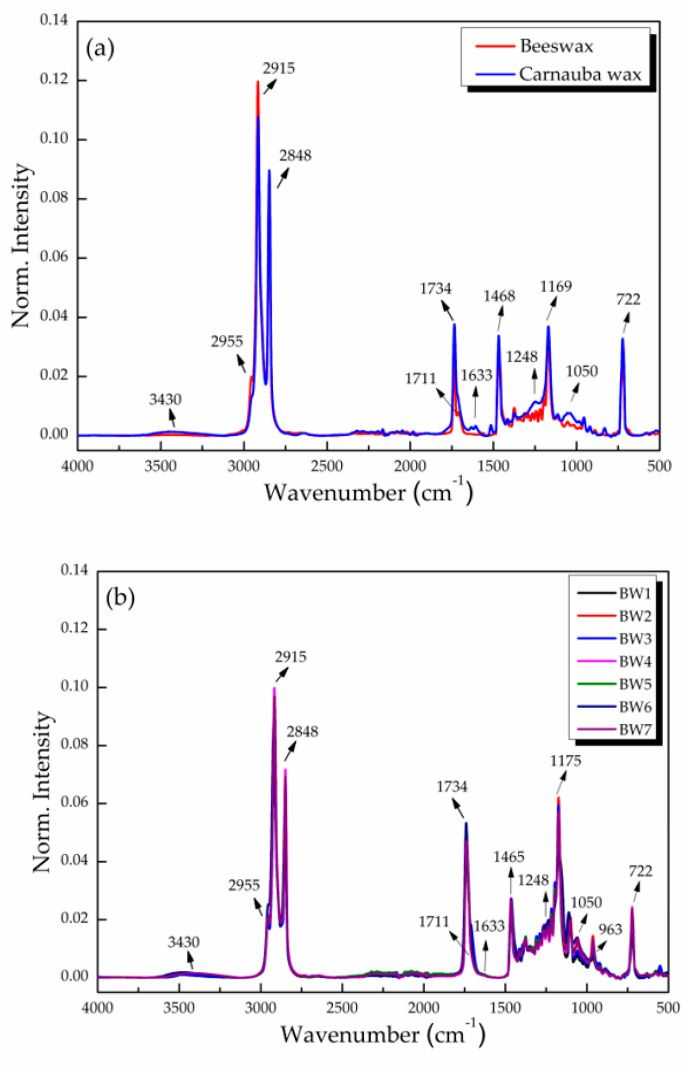
Normalized FTIR–ATR spectra of (**a**) natural waxes (beeswax and carnauba) and (**b**) biowaxes BW1–BW7 extracted from palm oil, with their respective designations of functional groups in the bands shown.

The FTIR analyses of beeswax and carnauba identified the presence of prominent peaks at 2915 and 2848 cm^−1^ (-CH), 1468 cm^−1^ (-CH_2_), 1373 cm^−1^ (-CH_3_), 3430 cm^−1^ (-OH), 1711 cm^−1^ (C=O), and 1734 cm^−1^ (C=O), indicating the presence of alkanes, alcohols, and carboxylic groups of carboxylic acids and esters, respectively. The signal at 1633 cm^−1^ corresponded to unsaturation (C=C); those at 1248, 1169, 1118, and 1050 cm^−1^ to ester group vibrations (C-C-O); and 722 cm^−1^ to long-chain alkanes (Figure 2a) [31]. The biowaxes studied (BW1–BW7) showed vibrations that were similar to the control waxes, with the intensity of their signals varying for some; i.e., they were lower at 2915 and 1734 cm^−1^ and practically null at 1633 cm^−1^, but were seen at 963 cm^−1^, which relates to the bending vibration of -HC=CH- (di-substituted trans-olefins) (Figure 2b).

### 3.5. UV–Vis Absorption Spectra

**Figure 3:**
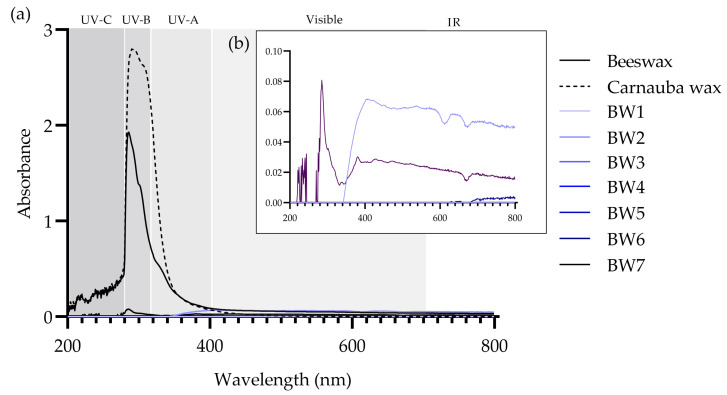
UV–Vis absorption spectra of (**a**) beeswax, carnauba wax, and biowaxes BW1–BW7 dissolved in toluene and measured at 20 °C, seen in the range of 200 to 800 nm, and (**b**) detail of the absorbances (0.00–0.10) of BW1–BW7 biowaxes.

BW1–BW7 diluted in toluene presented low-intensity bands or no bands in the 200- to 800-nm range (Figure 3). In contrast, the spectra of beeswax and carnauba wax showed absorption peaks in the 200- to 400-nm UV range, with maxima at 290 and 285 nm, respectively (Figure 3a). These peaks could be related to the presence of some of the di-esters of cinnamic acid that are found in carnauba wax and certain beeswax compounds, some of which are being proposed as sunscreens [32].

### 3.6. ^1^H NMR Analysis

**Figure 4:**
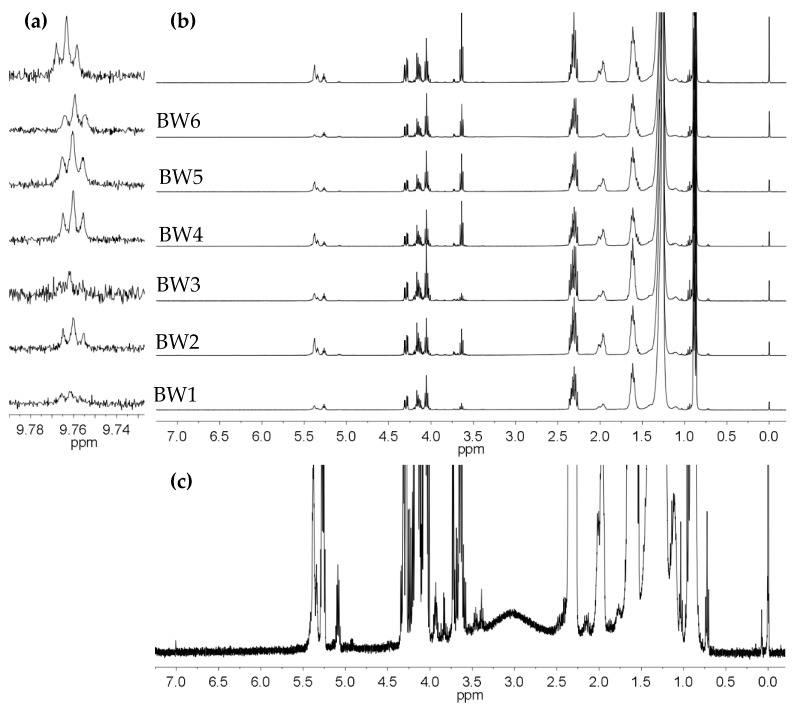
The 400-MHz ^1^H NMR spectra of the biowaxes obtained from hydrotreating refined and bleached palm and palm kernel oils (BW1–BW7): (**a**) broader spectrum in the region of the n-alkanals, (**b**) region of the CH_3_, CH_2_, and CH groups, and (**c**) broader spectrum of the BW6 biowax, where the resonances and multiplets of some of the minority species can be seen.

Figure 4b presents the ^1^H NMR spectra of biowaxes BW1–BW7. As shown, their compositions are similar, varying only in the intensity of their signals and the concentrations of each of their compounds. The protons are in the range of 0.6 to 1.06 ppm, which corresponds to terminal CH_3_ groups. These belong to the saturated or unsaturated alkyl chains of structures such as the paraffins or fatty acids that are in glycerides, esters, and alcohols. The different types of CH_2_ multiplets can be seen in the region 1.06 to 4.77 ppm, where the signal predominates at 1.26 ppm, which corresponds to methylene groups in structures that are part of linear alkyl chains, such as R(CH_2_)_n_-R. Regarding CH groups, these appear from 4.87 to 6.92 ppm and are part of the glyceryl group in mono-, di-, and triglycerides or unsaturated fatty acid chains. Functional groups were found in biowaxes BW1–BW7 in low concentrations as linear aldehyde groups (9.47 to 9.78 ppm), which are secondary oxidation products (Figure 4a). A detailed presentation of each type of proton that was identified in the NMR spectra of the different biowaxes was produced based on previous studies that used hydrogen NMR with different types of compounds found in vegetable oils [33,34,35]. Minority compounds were found when the spectra were magnified (Figure 4c).

The average number of carbon atoms per alkyl chain was determined with ^1^H NMR using the equation $n_{C=O}=\left(A_{0-3.33}+A_{5.31-6.92}\right)/A_{2.22-2.39}$, where $A_{0-3.33}$ represents the normalized area of the region between 0 and 3.3 ppm, which corresponds to alkyl chain hydrogens belonging to CH, CH_2_, and CH_3_ groups, while $A_{5.31-6.92}$ corresponds to the value of the normalized area of the region between 5.31 and 6.92 ppm, indicating the presence of unsaturated alkyl CH groups. The region 2.22 to 2.39 ppm represents protons at the α-position to the carbonyl group of carboxylic acids or esters that are in free fatty acids (FFA), waxy esters (WE), and glycerides (MG, DG, and TG). The average number of carbons per alkyl chain was thereby determined for the samples of RPO, BPO, BW1, BW2, BW3, BW4, BW5, BW6, and BW7, resulting in values of 16.30, 16.18, 23.82, 21.03, 22.86, 26.49, 25.53, 19.52, and 24.28, respectively. This parameter increased between 5 and 10 carbon atoms after the hydrotreating reactions. For the biowax that was derived from bleached palm oil (BW7), the average alkyl chain length increased by 8 carbon atoms. Meanwhile, the BW6 biowax obtained from refined palm kernel oil presented the lowest $n_{C=O}$ value, with 19.52 ± 1. Lastly, the values found for $n_{C=O}$ did not directly correlate with the average melting point values reported in Table 2. Therefore, not only does the melting point increase with the increase in the alkyl chain, but the existence of bulky compounds such as glycerides increases the number of van der Waals interactions per unit of weight, thereby influencing this property.

### 3.7. Microbiologic Test

None of the biowaxes evaluated presented growth of microorganisms in the BHI medium, and no bacteria or fungi were detected in the media at the times and temperatures used.

### 3.8. Cytotoxicity Tests

**Figure 5:**
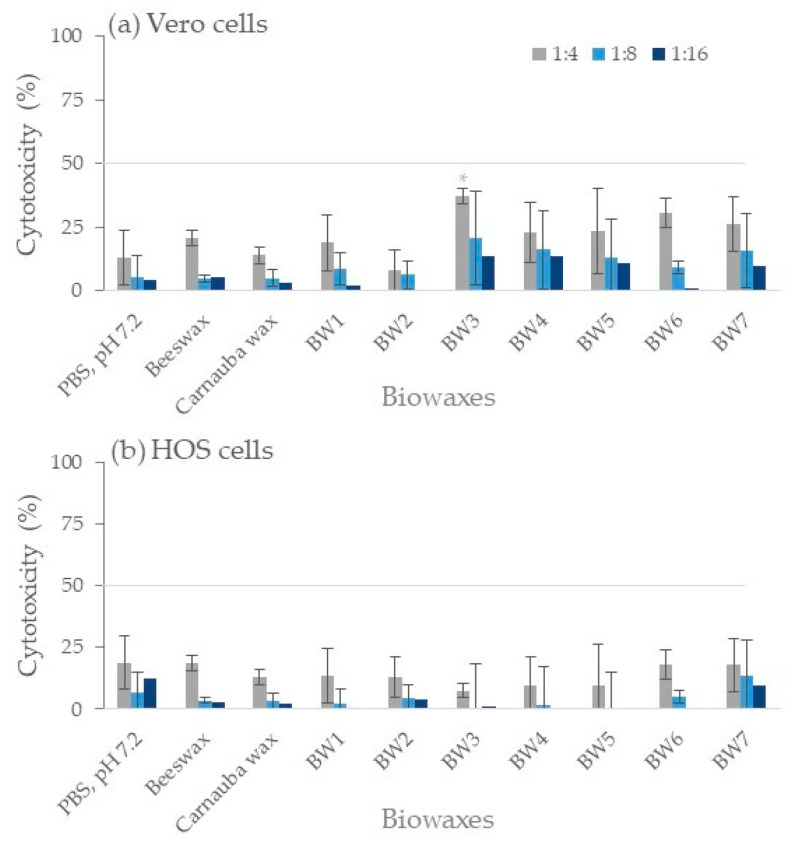
Cytotoxicity tests in Vero cells (**a**) and HOS cells (**b**). A bar graph shows the toxic effect of the supernatant obtained after 20 days of incubation with each wax. The control (shown as PBS, pH 7.2) was incubated without wax for 20 days under the same conditions. Lower cytotoxicity values than 50% (gray line) were considered non-toxic in this work. The experiment was repeated in triplicate (*n* = 9). * *p* < 0.05.

The 1:2 dilutions of the BW1–BW7 biowax supernatants presented toxicities of 43% to 66% in Vero cells and 34% to 54% in HOS cells. Beeswax presented 60.1% in Vero and 71.9% in HOS cells, and carnauba wax showed 43% in Vero and 38.6% in HOS cells. Since the vehicle used (PBS) also presented slight toxicity (30% to 40%) in both cell lines, the supernatant was considered cytotoxic when cytotoxicity was over 50% with a 1:4 dilution (Figure 5). Toxicity values at 1:4 dilutions of biowaxes BW1–BW7, beeswax, and carnauba wax supernatants were low, less than 37%. Statistical differences were obtained between PBS control and BW3 (*p* < 0.05); however, their toxicity values were less than 50%. Therefore, the biowaxes studied were not cytotoxic to the cell lines evaluated. In vitro findings of the non-cytotoxicity of some natural waxes (beeswax or carnauba) by studies that used lower concentrations (3.2 mg wax/mL) or supernatants of only 7 days (versus the 20 days used by the present study) were similar to the findings obtained by the work herein [36,37].

### 3.9. DPPH Radical Inhibition

**Figure 6:**
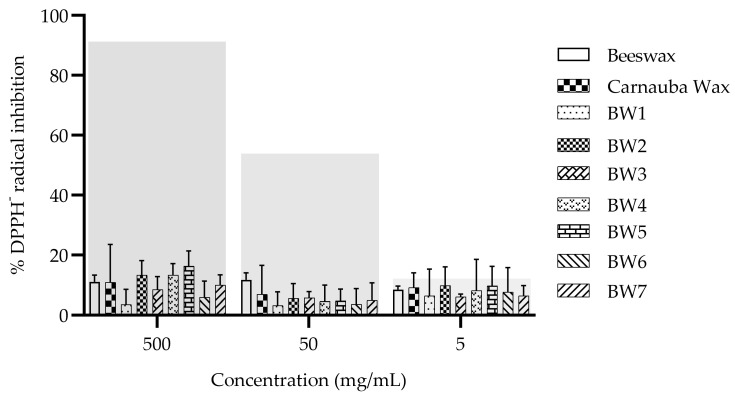
Antioxidant activity of the waxes that were derived from palm/palm kernel oil. Control: ascorbic acid. The gray transparent bars represent the percentage inhibition of the DPPH radical scavenging activity of ascorbic acid at 1, 0.1, and 0.01 mg/mL (left to right, respectively).

The BWs and controls studied did not exhibit antioxidant activity as values less than 20% of DPPH radical inhibition at all evaluated concentrations, without statistical differences (*p* > 0.05) between waxes and PBS control observed (Figure 6). In contrast, the ascorbic acid control reduced the DPPH radical, with an EC_50_ activity of 0.087 ± 0.003 mg/mL. Although palm fruits and palm oil extracts have shown antioxidant activity due to the presence of tocopherols, carotenoids, and phenolic compounds, among others [38,39], these could be lost in the processing that is used to produce biowaxes. Certain alcohol extracts and some of the components of carnauba wax (i.e., p-methoxycinnamic diesters) have shown antioxidant activity [17,40], as have honeycomb extracts, with an EC_50_ of 5.91 ± 0.27 mg/mL attributed to the higher flavonoid content [41].

### 3.10. Irritation (for Pharmaceutical Purposes Only)

**Figure 7:**
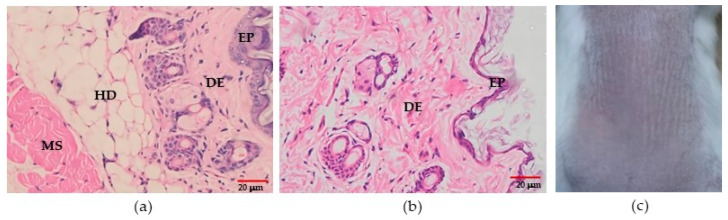
Irritation test. Representative microscopic and macroscopic features after 21 days of treatment. (**a**) Microphotography of skin after BW7 treatment and (**b**) skin treated with vehicle (magnification 400×). (**c**) Photography of the skin from the mice treated with BW7. HD: hypodermis. DE: dermis. EP: epidermis. MS: muscle.

During treatment of the experimental mice for 21 days and until 15 days post-treatment, no irritation resulted from the biowaxes (BW1–BW7) with a concentration greater than 30%, or from the vehicle without biowax. The histopathological study after treatment with BW2 showed mild acanthosis, spongiosis, and exocytosis in the epidermis, and mild acanthosis and exocytosis occurred with biowax BW7. However, this was also observed in the mice that were treated with the control vehicle (formulation without BW). No inflammatory reaction in the dermis was observed in any of the biopsies. In addition, two of the observers indicated that the biopsies of the irritated skin in the BALB/c mice that were treated with biowaxes were the same as those of the mouse that was treated with the mineral oil vehicle (Figure 7).

## 4. Conclusions

The BW1–BW7 biowaxes obtained by hydrotreating palm and palm kernel vegetable oils showed excellent physicochemical properties, including an intermediate hardness, which, when using this type of biowax, can eventually result in products with sensory characteristics related to their extensibility. It is also noteworthy that since these biowaxes contain fatty alcohols, their characteristics are different from those of carnauba waxes and beeswax, which do not have this type of chemical family. The biowaxes studied are non-cytotoxic, non-phototoxic, and non-irritating, so they can serve as suitable raw materials for products for human use.

## Figures and Tables

**Table 1:** Characteristics of biowaxes (BW).

BW	Origin	Photo	Color/ Estate	E	pH (Days)			MO	MP °C	PV mm
					5	10	20			
1	RPO	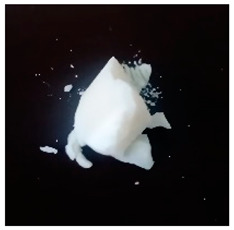	yellowish, solid	1	5.1	4.9	5.0	-	47.9	2.1
				2	4.9	4.9	4.8	-		
2	RPO	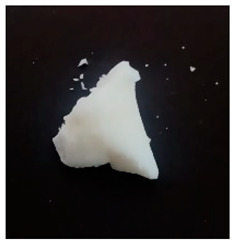	yellowish, solid	1	4.9	4.9	4.6	-	42.7	2.9
				2	5.0	5.1	5.0	-		
3	RPO	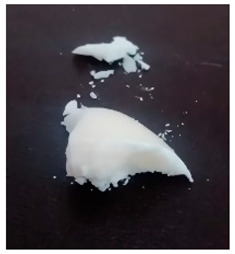	yellowish, solid	1	4.8	4.8	4.9	-	44.9	2.6
				2	4.9	4.8	4.8	-		
4	RPO	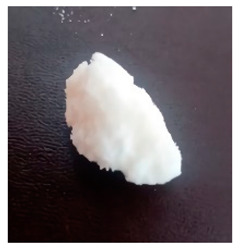	yellowish, solid	1	4.7	4.7	4.6	-	40.2	3.8
				2	4.8	4.7	4.8	-		
5	RPO	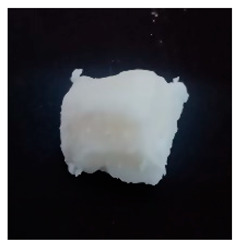	yellowish, solid	1	4.8	4.6	4.5	-	43.3	3.2
				2	4.7	4.6	4.6	-		
6	RPKO	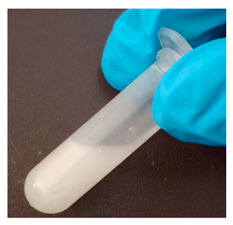	yellowish, liquid	1	4.5	4.3	4.4	-	<20	lí-quid
				2	4.6	4.5	4.6	-		
7	BPO	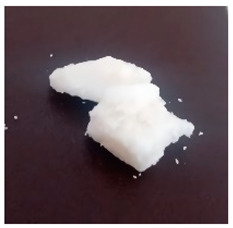	yellowish, solid	1	4.9	4.7	4.6	-	40.1	2.9
				2	5.1	5.1	4.9	-		

RPO: refined palm oil, RPKO: refined palm kernel oil, BPO: bleached palm oil, MO: microorganisms (bacteria or fungi), MP: average melting point, PV: penetration value.

**Table 2:** Composition of palm oil–based biowaxes [wt.%].

BC	Paraffin	FFA	FA	WE	MG/DGs	TG
Beeswax ^1^	11–28	0.8–18	0.4–1.8	57–74	-	-
Carnauba wax ^2^	1.5–3.0	3.0–3.5	2.0–3.0	84–85	-	-
Candelilla wax ^2^	50–51	7.0–9.0	-	28–29	-	-
BW1	31.32	15.33	5.79	25.39	0.77	21.40
BW2	28.40	8.80	2.10	23.70	2.00	28.40
BW3	15.18	13.76	5.45	35.64	1.42	28.57
BW4	40.00	11.10	2.30	26.70	1.30	18.60
BW5	13.30	14.50	13.50	34.50	3.70	20.50
BW6	27.90	27.40	10.80	16.70	5.50	11.80
BW7	29.60	14.30	7.60	24.80	1.70	22.00
RPO	-	6.10 ^b^	-	-	2.40 ^b^	91.50 ^b^

P: paraffin, FFA: fatty acids, FA: fatty alcohols, WE: waxy or fatty esters, MG: monoglyceride, DG: diglyceride, TG: triglyceride, AMP: average melting point, PV: penetration value, RPO: refined palm oil. Values reported by ^1^ Hepburn et al., 2014 and ^2^ Guzman et al., 2013 [9,29].

## Data Availability

Not applicable.

## Supplementary Materials

The following supporting information can be downloaded at: https://www.mdpi.com/article/10.3390/ma16124402/s1. Figure S1: Scanning electron microscopy (SEM) of biowaxes (a,b) BW1, (c,d) BW2, (e,f) BW3, (g,h) BW4, (i,j) BW5, (k,l) BW6, (m,n) BW7, (o,p) beeswax, and (q,r) carnauba wax with two magnification values between 100× and 400× (right) and 1500× and 3000× (left).

## References

[1] Tinto W.F., Elufioye T.O., Roach J. (2017). Waxes. Pharmacognosy.

[2] Grand View Research, Inc (2017). Paraffin Wax Market Analysis by Application (Candles, Packaging, Cosmetics, Hotmelts, Board Sizing, Rubber), by Region (North America, Europe, Asia Pacific, Central & South America, Middle East & Africa), by Country, and Segment Forecasts, 2014–2025.

[3] Yao L., Lio J., Wang T., Jarboe D.H. (2013). Synthesis and Characterization of Acetylated and Stearylyzed Soy Wax. J. Am. Oil Chem. Soc..

[4] Cameron T.C., Wiles D., Beddoe T. (2021). Current Status of Loop-Mediated Isothermal Amplification Technologies for the Detection of Honey Bee Pathogens. Front. Vet. Sci..

[5] Keng P.S., Basri M., Zakaria M.R.S., Rahman M.B.A., Ariff A.B., Rahman R.N.Z.A., Salleh A.B. (2009). Newly synthesized palm esters for cosmetics industry. Ind. Crop. Prod..

[6] Basri M., Abdul Rahman R.N.Z., Salleh A.B. (2013). Speciality oleochemicals from palm oil via enzymatic synthesis. J. Oil Palm Res..

[7] Chang S.H. (2013). Vegetable oil as organic solvent for wastewater treatment in liquid membrane processes. Desalin. Water Treat..

[8] Sambanthamurthi R., Sundram K., Tan Y.A. (2000). Chemistry and biochemistry of palm oil, Prog. Lipid Res..

[9] Guzmán M., Kafarov V., Guzmán A., Garzón L. (2013). Influence of temperature during crude palm oil hydrotreating over NiMo/γ-Al_2_O_3_ catalysts. Rev. ION.

[10] Masudi A., Muraza O. (2018). Vegetable Oil to Biolubricants: Review on Advanced Porous Catalysts. Energy Fuels.

[11] Malins K. (2021). Synthesis of renewable hydrocarbons from vegetable oil feedstock by hydrotreatment over selective sulfur-free SiO_2_-Al_2_O_3_ supported monometallic Pd, Pt, Ru, Ni, Mo and bimetallic NiMo catalysts. Fuel.

[12] Guzman A., Torres J.E., Prada L.P., Nuñez M.L. (2010). Hydroprocessing of crude palm oil at pilot plant scale. Catal. Today.

[13] FDA Food and Drugs Chapter I—Federal Food, Drug, and Cosmetic Act, Part 184, Direct FOOD Substances Affirmed as Generally Recognized as Safe, Sec. https://www.accessdata.fda.gov.

[14] Iyisan B., Simon J., Avlasevich Y., Baluschev S., Mailaender V., Landfester K. (2022). Antibody-Functionalized Carnauba Wax Nanoparticles to Target Breast Cancer Cells. ACS Appl. Bio Mater..

[15] Soleimanian Y., Goli S.A.H., Shirvani A., Elmizadeh A., Marangoni A.G. (2020). Wax-based delivery systems: Preparation, characterization, and food applications. Compr. Rev. Food Sci. Food Saf..

[16] De Souza I.D.L., Saez V., De Campos V.E.B., Nascimento M.R., Mansur C.R.E. (2020). Multiple Response Optimization of Beeswax-Based Nanostructured Lipid Carriers for the Controlled Release of Vitamin E. J. Nanosci. Nanotechnol..

[17] de Freitas C.A.S., de Sousa P.H.M., Soares D.J., da Silva J.Y.G., Benjamin S.R., Guedes M.I.F. (2019). Carnauba wax uses in food—A review. Food Chem..

[18] Carrillo J.-C., Danneels D., Woldhuis J. (2021). Relevance of animal studies in the toxicological assessment of oil and wax hydrocarbons. Solving the puzzle for a new outlook in risk assessment. Crit. Rev. Toxicol..

[19] Bruusgaard-Mouritsen M.A., Johansen J.D., Zachariae C., Kirkeby C.S., Garvey L.H. (2020). Natural ingredients in cosmetic products—A suggestion for a screening series for skin allergy. Contact Dermat..

[20] Nyman G.S.A., Tang M., Inerot A., Osmancevic A., Malmberg P., Hagvall L. (2019). Contact allergy to beeswax and propolis among patients with cheilitis or facial dermatitis. Contact Dermat..

[21] EFSA Panel on Food Additives and Nutrient Sources added to Food (ANS) (2012). Scientific Opinion on the re-evaluation of carnauba wax (E 903) as a food additive. EFSA J..

[22] Fiume M.M., Heldreth B., Bergfeld W.F., Belsito D.V., Hill R.A., Klaassen C.D., Liebler D.C., Marks J.J.G., Shank R.C., Slaga T.J. (2015). Safety Assessment of Alkyl Esters as Used in Cosmetics. Int. J. Toxicol..

[23] Burnett C.L., Fiume M.M., Bergfeld W.F., Belsito D.V., Hill R.A., Klaassen C.D., Liebler D., Marks J.G., Shank R.C., Slaga T.J. (2017). Safety Assessment of Plant-Derived Fatty Acid Oils. Int. J. Toxicol..

[24] González-Muñoz P., Conde-Salazar L., Vañó-Galván S. (2014). Dermatitis alérgica de contacto a cosméticos. Actas Dermosifiliogr..

[25] Panico A., Serio F., Bagordo F., Grassi T., Idolo A., De Giorgi M., Guido M., Congedo M., De Donno A. (2019). Skin safety and health prevention: An overview of chemicals in cosmetic products. J. Prev. Med. Hyg..

[26] Olarte G., Garzón L., Sarmiento J., López-Giraldo L.J., Vivas-Báez J.C. (2023). Biowax Production from the Hydrotreatment of Refined Palm Oil (RPO). Processes.

[27] Murillo-Méndez C., López-Giraldo L.J., Ramírez Quintero A.F., Castañeda-Rodas M. (2022). Planteamiento de un modelo matemático de características macroscópicas de bioceras producidas del aceite de palma con interés comercial. Rev. ION.

[28] Fei T., Wang T. (2017). A review of recent development of sustainable waxes derived from vegetable oils. Curr. Opin. Food Sci..

[29] Hepburn H.R., Pirk C.W.W., Duangphakdee O. (2014). The Chemistry of Beeswax. Honeybee Nests.

[30] Trisnadewi T., Kusrini E., Nurjaya D.M., Putra N., Mahlia T.M.I. (2021). Experimental analysis of natural wax as phase change material by thermal cycling test using thermoelectric system. J. Energy Storage.

[31] Rivera-Barrera D., Rueda-Chacón H., Molina D. (2020). Prediction of the total acid number (TAN) of colombian crude oils via ATR–FTIR spectroscopy and chemometric methods. Talanta.

[32] Villalobos J.R., Müller C.C. (2006). Sun protection enhancement of titanium dioxide crystals by the use of carnauba wax nanoparticles: The synergistic interaction between organic and inorganic sunscreens at nanoscale. Int. J. Pharm..

[33] Shi T., Zhu M., Zhou X., Huo X., Long Y., Zeng X., Chen Y. (2019). 1H NMR combined with PLS for the rapid determination of squalene and sterols in vegetable oils. Food Chem..

[34] Jęczmionek L., Krasodomski W. (2015). Biocomponents via Zeoforming and Hydroconversion of Vegetable Oil: ^1^H NMR Analysis of Glycerides Conversion. Energy Fuels.

[35] Calò F., Girelli C.R., Angilè F., Del Coco L., Mazzi L., Barbini D., Fanizzi F.P. (2021). 1H-NMR Profiling Shows as Specific Constituents Strongly Affect the International EVOO Blends Characteristics: The Case of the Italian Oil. Molecules.

[36] Zhang Y., Bi J., Wang S., Cao Q., Li Y., Zhou J., Zhu B.-W. (2019). Functional food packaging for reducing residual liquid food: Thermo-resistant edible super-hydrophobic coating from coffee and beeswax. J. Colloid Interface Sci..

[37] Wang W., Lockwood K., Boyd L.M., Davidson M.D., Movafaghi S., Vahabi H., Khetani S.R., Kota A.K. (2016). Superhydrophobic Coatings with Edible Materials. ACS Appl. Mater. Interfaces.

[38] Ong A.S.H., Goh S.H. (2002). Palm Oil: A Healthful and Cost-Effective Dietary Component. Food Nutr. Bull..

[39] Balasundram N., Ai T.Y., Sambanthamurthi R., Sundram K., Samman S. (2005). Antioxidant properties of palm fruit extracts. Asia Pac. J. Clin. Nutr..

[40] Silva C.A., Pinto I.G., Machado P.H., Rodrigues C., Costa M.L., Florindo M.I. (2016). Carnauba wax p-methoxycinnamic diesters: Characterisation, antioxidant activity and simulated gastrointestinal digestion followed by in vitro bioaccessibility. Food Chem..

[41] Zhao H., Zhu M., Wang K., Yang E., Su J., Wang Q., Cheng N., Xue X., Wu L., Cao W. (2019). Identification and quantitation of bioactive components from honeycomb (Nidus Vespae). Food Chem..

