# Supplementary material for: Biowaxes from Palm Oil as Promising Candidates for Cosmetic Matrices and Pharmaceuticals for Human Use
Source: Materials (Basel). 2023 Jun 15;16(12):4402. doi: 10.3390/ma16124402 (PMC10301727; doi:10.3390/ma16124402)
Supplement: Supplementary file 1 [file materials-16-04402-s001.zip › materials-2392857-supplementary.pdf]

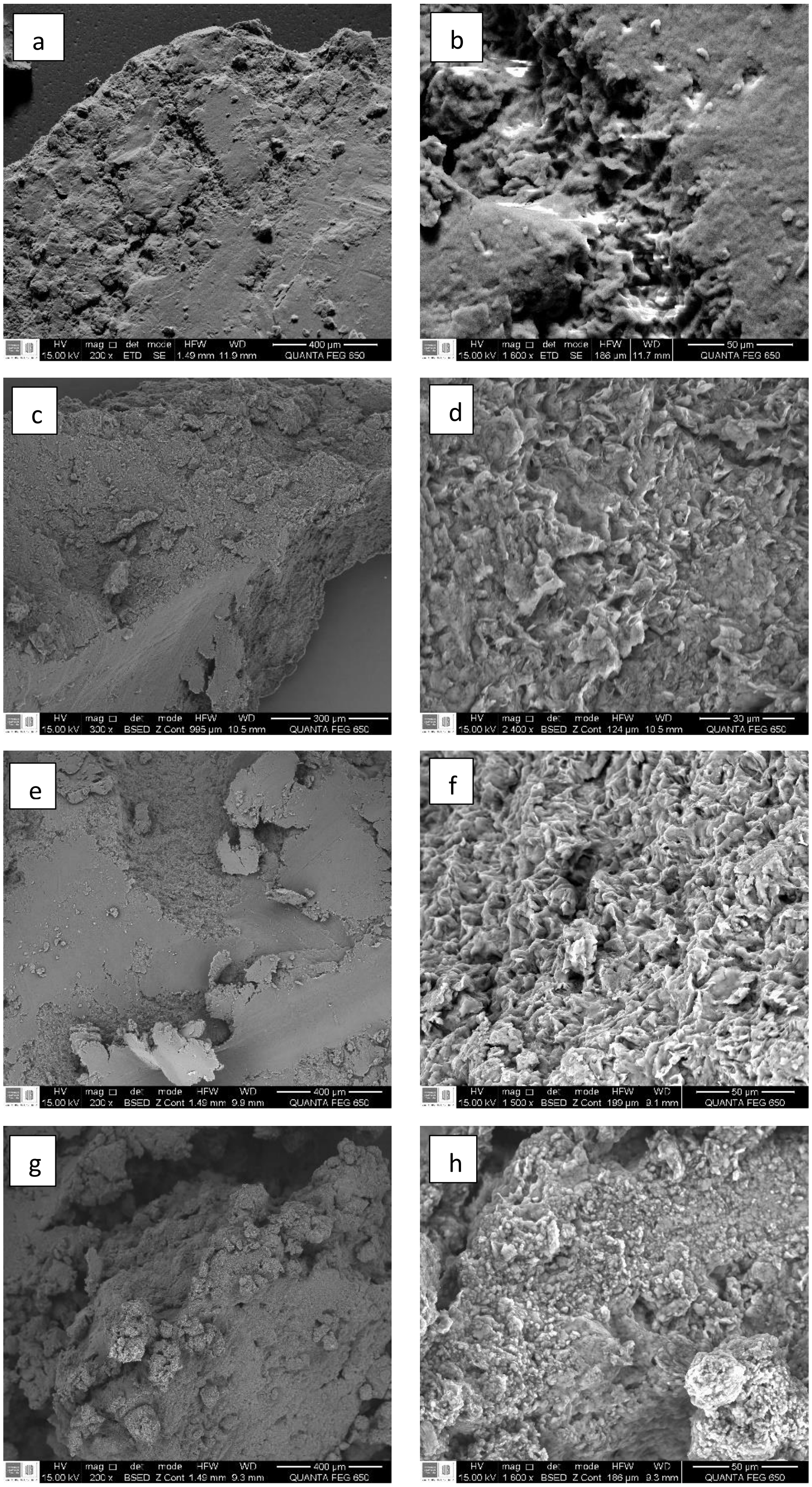

a
400 µm
b
50 µm
c
300 µm
d
30 µm
e
400 µm
f
50 µm
g
400 µm
h
50 µm
QUANTA FEG 650

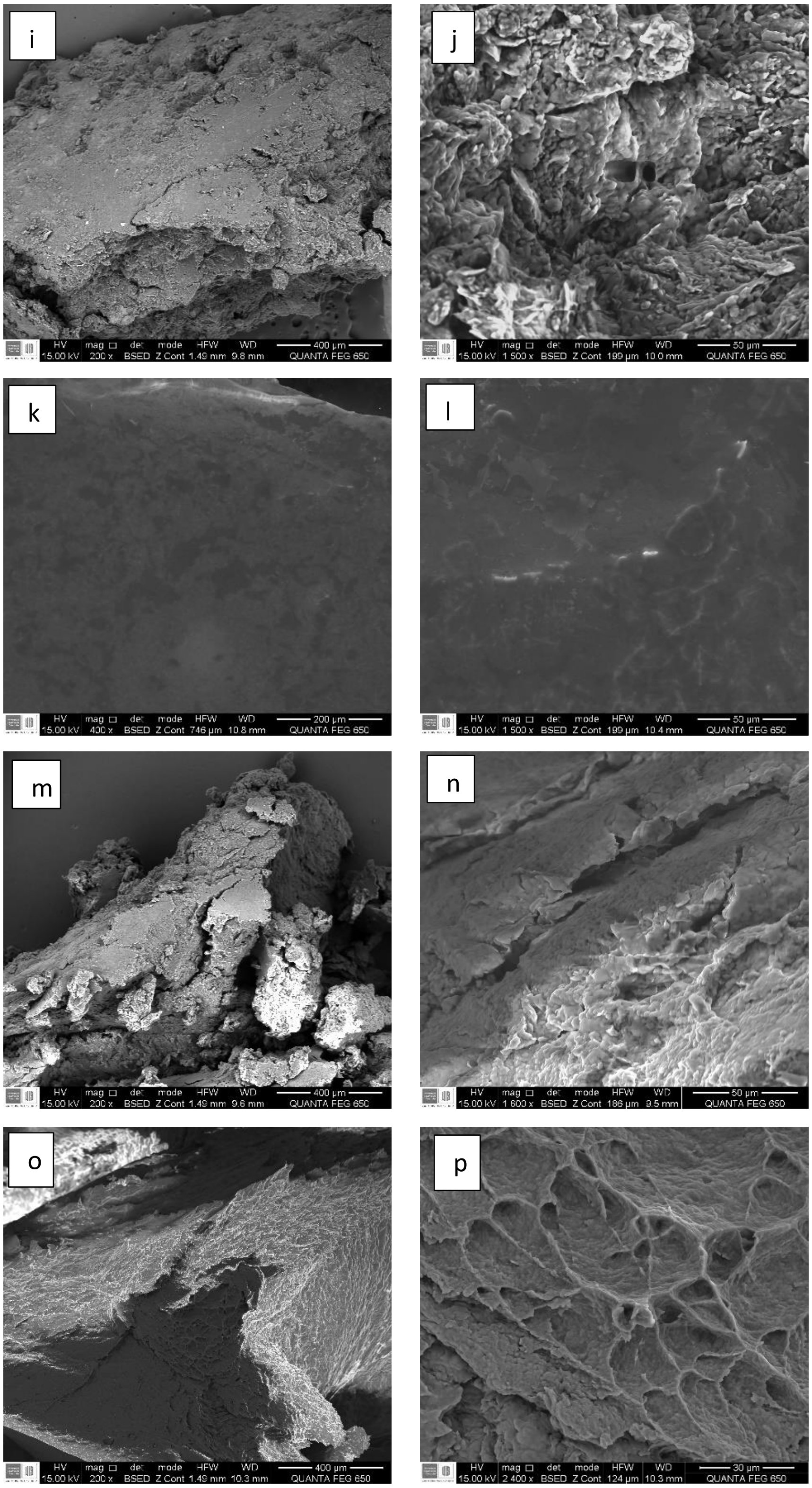
i
j
k
l
m
n
o
p

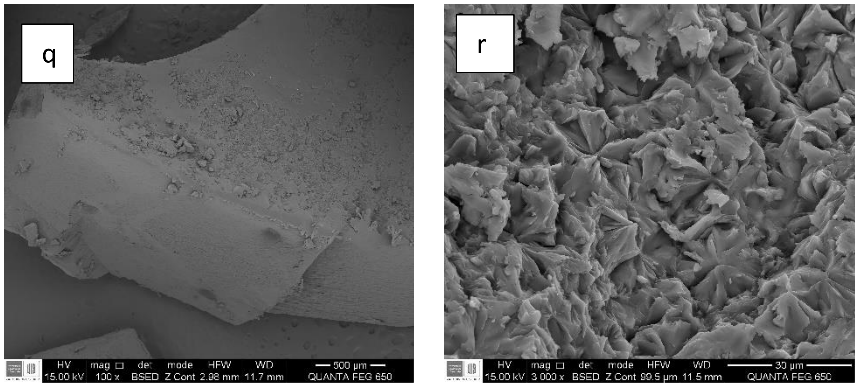

Figure S1. Scanning electron microscopy (SEM) of biowaxes (a-b) BW1, (c-d) BW2, (e-f) BW3, (g-h) BW4, (i-j) BW5, (k-l) BW6, (m-n) BW7, (o-p) beeswax and (q-r) carnauba wax with two magnification values between 100-400x (right) and 1500-3000x (left).
